# Two Cases of Strangulated Hernia—Operation—Radical Cure in One Case

**Published:** 1867-08

**Authors:** J. R. Lothrop


BUFFALO

### Medical and Surgical Journal.

VOL. VII. AUGUST, 1867. No. 1.

### Original Communications.

ART. I.—*Two cases of Strangulated Hernia — Operation — Radical cure in one case.* By J. R. Lothrop, M. D.

Case 1.—In this case a young man who had been afflicted with inguinal hernia about three years, was subjected to an operation for the relief of a strangulation which had existed about three days — from Sunday to Wednesday following. The hernia, as stated, was generally wholly and easily reduced, and the bowel effectually kept back by a truss. Before the patient came under my observation, taxis had been very thoroughly practiced. I did not, therefore, think it advisable to make an effort at reduction. The symptoms of strangulation were present, viz: a tumor painful to the touch, abdominal pain, vomiting, small quick pulse, cold moist skin, anxious countenance, and restlessness.

An operation being deemed proper, it was immediately decided upon. Assisted by Drs. Pratt, Garvin, and Sheldon, chloroform being given, I made the operation by incision of the sac. The contents of the sac were found to be omentum and intestine. The first portion was omental and irreducible. Behind this was the incarcerated intestine. After a slight incision of the ring the intestine was retured. It was of a dark claret color. Having effected this, a question arose as to what it was best to attempt with the protruding omentum. It was found to be adherent to the sac, which again was attached in the scrotum, and could not be returned without tearing the adhesions. This was deemed the best course. The adhesions were torn away and the omental portion returned. Before this was done, however, there was some delay to arrest bleeding, which was quite free. Two silk ligatures were applied, one cut short and the other left long, so as to hang out at the external incision—this last, however, by inadvertence, as my intention was to cut both short.

In closing the wound the following method was adopted:—Two deep sutures of silver were inserted, one near the ring and the other three-fourths of an inch lower. The sutures were passed deeply with the intent of including most of the neck of the sac, the finger being passed into the inguinal canal as a guide. These sutures were allowed to remain a week or more, and were then removed. The object aimed at was closure of the neck of the sac, and hence radical cure. This object seemed, for a time, secured. The young man recovered from the operation without any bad symptoms, and was soon about. A hard mass could be felt about the ring, and apparently formed in part, by the adhesion of the returned omental mass over the opening of the inner abdominal ring. As stated above, a radical cure seemed effected. There was no descent of the intestine for four months, though a truss was worn most of the time. After that time, however, a small knuckle of intestine was forced down by an unusual exertion. A gradual increase in the bulk of intestine, at each descent, followed. At the present time the hernia, when not restrained by a truss, is as large as before the operation, but it appears to be wholly intestine, no omentum escaping.

Case 2.—A colored boy, about 14 years of age, was admitted to the Buffalo General Hospital, with what was thought to be a strangulated congenital hernia. As is usual in such cases, previous to admission, taxis had been made pretty thoroughly, and the tumor was painful when handled. Incarceration had existed several days. The usual symptoms of prolonged strangulated hernia were present, viz: vomiting, anxious countenance, small quick pulse, cold sweating. It was evident from examination of the tumor, after anæsthesia was produced, that no decided indications of the presence of intestine were present. There was evidently fluid in the sac. Assisted by Dr. Sheldon I made the operation soon after the reception of the patient. The incision of the sac was followed by the escape of two or three ounces of bad-smelling, greenish fluid. The neck of the sac was obstructed by a soft mass which the finger pushed before it into the abdomen. This was either intestine or omentum, and may have been the strangulated portion. The sac was one-fourth of an inch in thickness. In this case the same measure was adopted for closure of the neck of the sac by deep silver sutures. Serious trouble followed the operation—extensive peritonitis—sloughing of the sac, and great suppuration. The condition of the patient was such for two or three weeks, as to excite doubt as to the result. After that time improvement took place. In about five weeks he was about the ward. In this case the cure was radical. The neck of the sac was closed, and the intestine did not and has not again descended. In the treatment of the peritonitis, hypodermic injection of morphia was employed and its value fully experienced.

*Remarks.*—It will be observed in the first case, that no apparent trouble followed the return of the torn and bleeding omentum with two ligatures attached. The long one came away in due time without exciting much suppuration. That cut short remained in the abdomen. In regard to the omental mass, there was a question whether to excise or return it. The latter was decided upon. Whether this procedure is preferable to excision is a matter of doubt. If the omental protrusion is excised, the portion left in the neck, by inflammation and adhesion may close it, and thus bring about a radical cure, but sensations of dragging and uneasiness follow such a course. The return of the omental mass is preferred by most surgeons. Leaving the omental mass unreduced exposes the patient to fresh danger of strangulation by escape of the intestine through the small opening which would remain. This liability also would attend the excision of the protruded portion, if the closure should not be entire. A small opening would be left into which the intestine would most surely be forced, and the occurrence of strangulation likely to take place. In that case, the measures taken to prevent the descent of the intestine, would serve to render the return more difficult.

In the second case the strangulation was not very well made out, and was somewhat doubtful. The symptoms of strangulation, or many of them, may be present without actual strangulation. But when the symptoms are present, both general and local, it is safest to presume its existence, and adopt treatment adapted to it. It may be that the whole trouble was inflammation of the hernial sac, though the symptoms were those of strangulation. In both cases, care was taken to avoid the vessels of the spermatic cord, and the testicle was not injured.

*Radical Cure.*—From the earliest periods various operations have been performed to close the inguinal canal, and thus prevent the re-descent of the intestine. The ancients applied the *cautery* to the skin over the ring, often deeply. Monro modified it by making an incision first, and then applying the cautery. If this treatment did not cause fatal results, it was not certain to procure the object aimed at, though it really effected some cures. This method, which no one would now practice, was employed till quite modern times.

*Ligatures*, including the integuments and the neck of the sac, and tied tightly, have been used. This method, though effectual in many cases, destroyed the testicle, and probably for that reason was abandoned. Celsus and Paulus Ægineta used the ligature. A mode similar to this called the "*punctum aureum*" was practiced by Ambrose Paré. By this method, a golden wire was passed under the neck of the sac, which was laid bare by incision, and tightened from time to time. The design of this was to partially close the canal and save the testicle. Though in some cases it succeeded, in most it did not effect its object, but sacrificed the testicle.

*The method by suture*, called "the royal stitch," because it did not deprive the subject of the power to increase the king's subjects, though better than the other means mentioned, fell into disuse, and was even condemned by surgeons. It was done in two ways; first, without incision, the needle being carried through the integuments and through the neck of the sac, and a sort of continuous suture made. Secondly, the neck of the sac was laid bare and the continued suture applied directly to it. This method was not always effectual, was looked upon as severe and dangerous, and was therefore condemned. It is not easy to understand why this method should have met with so severe a reprobation by many modern surgeons, for subcutaneous suture is one of the features of the most popular, and upon the whole most successful method practiced at the present time—that of Mr. John Wood, King's College, London. In the cases above related the method of suture was employed, including a part of the neck of the sac and a small portion of the integument, but saving the vessels of the spermatic cord. This method by suture is only applicable to inguinal hernia.

*Excision of the sac*, either wholly or in part, was another method employed sometimes with success, yet in many cases followed by death. In regard to this method, it is probably true that the disfavor into which it fell, was as much due to the unskillfnl manner in which it was performed, as to any want of merit in the operation itself. In certain cases the operation is followed by good results.

*Incision of the sac* was practiced so late as 1832. An incision was made from the neck to the bottom of the sac, and portions on each side removed. This was a severe and dangerous, as well as uncertain method.

*Castration*, as a method of cure, less dangerous and less effectual, was, we may hope, mostly practiced by ignorant charlatans, and is not to be spoken of as a method deserving anything but condemnation. This is true in the main of the method of closing the ring by forcing and retaining the testicle in it, except that it is more dangerous. The testicle, probably, can neither be made to remain in the ring nor entirely close it.

I may mention some of the operations which have been practiced by individual surgeons of late years, only to show the great variety of proceedures resorted to. They bear evidence to the recognized importance of curing an infirmity which has occupied the thoughts of surgeons from the earliest periods—from Celsus downwards to the present.

In modern times, PETIT attempted to close the inguinal canal by crowding the sac itself into it, with the hope that adhesion would take place strong enough to resist the descent of the bowel. An opening was first made down to the sac.

GERDY on the other hand attempted closure of the canal by crowding and stitching the integuments into it and removing the cuticle by caustic alkalies. This was called "invagination by the integuments," and is the basis of several recent and rather popular operations.

BELMAS' operation consisted in crowding a pouch or roll of goldbeater's skin into the upper part of the sac, and afterwards into the neck of the sac near the ring, thus attempting the closure by causing inflammation and adhesion.

GRAEFE made an incision at the external ring, cut off the neck of the sac, and crowded a roll of lint into the inguinal canal as far as the internal ring, with the expectation of causing inflammation and closure by adhesion. The introduction of a seton into the neck of the sac for the purpose of closing it, is essentially the same proceeding. This method, rough as it was, effected some cures. The operation of plugging the inguinal canal by leaving the omentum in it, after its protruding portion had been cut off, in the operation for strangulated hernia, was a better and more successful proceeding, though similar in design. Cooper and Velpeau succeeded by this method.

BONNET used pins to bring about the requisite inflammation. The pins were introduced beneath the skin and near the ring, one above and the other below the neck of the sac, in such a manner that the neck could be compressed between them, by bending one over the other, and thus excite inflammation in from six to twelve days.

MAYOR used needles instead of pins, thus carrying a ligature around the neck of the sac and tying it over a piece of sponge. The ligature could be tightened from time to time and allowed to remain long enough to produce the desired effect.

VELPEAU scarified the inguinal canal, a proceeding not devoid of danger from hemorrhage, and Guerin scarified the neck of the sac by a subcutaneous method. Similar to this in idea was the method by acupuncture of the neck of the sac near the external ring, advocated by Malgaine, and practiced considerably in this country and in Europe.

VELPEAU and DR. PANCOAST practiced injection of iodine or tincture of cantharides, in or near the neck of the sac. The former opened the neck and injected the iodine into it. The latter introduced it subcutaneously by means of a small syringe. It is difficult to hit the sac by subcutaneous puncture.

It will be seen that the object aimed at in all the operations related above was closure or contraction of the neck of the hernial sac. Even Velpeau's proceeding of scarifying the inguinal canal, seems to have no further design than to close the sac in the canal. These operations failed in most cases to bring about a closure of sufficient resisting power to prevent the descent of the intestine, and for the reason as stated by Lawrence that "something more is required; we want a remedy that should contract the tendinous opening; for while that remains preternaturally large a new protrusion is a highly probable occurrence."

Admitting that the operations accomplish all they are designed to do, viz: close completely the neck of the sac, they will not contract the tendinous opening, and hence a new sac may be formed even if the closure of the old sac is perfect, and thus the intestine protrude anew. In order that the case then shall be truly radical, some measure should be devised to close or contract the ring itself. This has been attempted. First, scarifying the ring, an old operation, but not effectual, and not free from danger of wounding the epigastric artery. Second, closing the ring, as practiced by Dr. Thomas Wood of Cincinnati, by means of sutures. The object is to close or constrict the external ring by a tendinous growth, for he remarks "tendons when wounded will unite again by a formation similar to their original structure." Theoretically this operation has an advantage over all others, but it does not seem to have been much employed, and the presumption is, that it has been found no more successful than many other methods.

All the methods described have been successful in some cases, some in a greater proportion than others. The two operations most popular at this time seem to be Wützer's and Mr. John Wood's. The first is essentially the old method of Gerdy, by invagination, the difference consisting in means devised to retain the invaginated integuments in the ring, viz: by means of an instrument. The second is essentially an attempt to close or contract the inguinal canal by means of subcutaneous suture or ligature. It is essentially the old idea of ligature or suture revived, but so applied as to obviate some of the objections to the old method, especially the destruction of the testicle. Invagination of the spermatic fascia and the hernial sac is also a feature of the operation. It is, therefore, a revival and combination of the old method by suture, and the method of Petit, viz: of crowding the sac into itself. Moreover, the ligatures are so applied as to lessen the tendinous opening. It therefore aims to effect a cure by combining the several methods; of dealing with the sac; of invaginating tissue into the inguinal canal; and of contracting the tendinous opening.

The first method, Wützer's, is theoretically a method of closing the external ring. Its object is to place a firm substance in the ring and fix it there by adhesion. The operation is very likely to fail, the adhesion of the invaginated portion not being so firm but that the intestine will push down. It fails because the scrotum by its weight draws out the invaginated portion, because it enlarges the external ring by crowding the integument in, and because adhesion does not take place at the posterior part of the canal, thus giving the intestine a chance to escape behind it.

The method of Mr. John Wood he claims has succeeded in many cases; patients having been kept under observation long enough to establish the fact of cure. The cases cured are in the proportion of seventy per cent. to the operations, a ratio of success much greater than has been attained by any other method. It has also this additional claim to attention, that it is a safe operation. Mr. Wood reports one death in one hundred and fifty operated upon by him. It is necessary to wait more than a year in an adult, and especially one at all advanced in years before deciding that the cure is radical. In children and youth the necessity for waiting for time to pass is less, and the probabilities of radical cure greater as time passes and the protrusion does not reappear.

The method by injection was once said to fail altogether. But time has shown that some cases treated by that method were permanently cured, and it will succeed in a certain proportion, the younger the subject the better the chances, which in fact, may be said of all methods. The method is more likely to succeed if the sac is scarified by a tenotomy knife before the iodine is injected, thus in a measure reviving Guerin's method of dealing with the neck of the sac by a subcutaneous scarification, and adding injection to it.

For an infirmity so common—about eight per cent. of the human family being afflicted with it—so inconvenient, and in some instances so dangerous, it is to be hoped some effectual remedy will soon be discovered, since all methods thus far practiced fail in a number of cases. We may express the hope that the new method promised by a surgeon of Philadelphia—Dr. D. Hayes Agnew—may prove that effectual one, which all surgeons have been anxious to find. Up to this time Wood's operation seems to have afforded most relief and is in most repute.

